# Comparative effects of granulocyte-colony stimulating factor and colistin-alone or in combination on burn wound healing in *Acinetobacter baumannii* infected mice

**Published:** 2018-12

**Authors:** Somayeh Soleymanzadeh Moghadam, Zeinab Fagheei Aghmiyuni, Hassan Zaheri, Nastaran Arianpour, Mohammad Reza Danaeifard, Maryam Roham, Mahnoush Momeni

**Affiliations:** 1 Antimicrobial Resistance Research Center, Institute of Immunology and Infectious Diseases, Iran University of Medical Sciences, Tehran, Iran; 2 Department of Microbiology and Immunology, Faculty of Medicine, Kashan University of Medical Sciences, Kashan, Iran; 3 Faculty of Veterinary Medicine, Islamic Azad University, Garmsar branch, Garmsar, Iran; 4 Burn Research Center, Iran University of Medical Sciences, Tehran, Iran

**Keywords:** Burn, Granulocyte-colony stimulating factor, Colistin, *Acinetobacter baumannii*, Wound infection

## Abstract

**Background and Objectives:**

Burn wounds are one of the most important health problems all over the world because infection after burn can delay wound healing. Treating burn wounds with granulocyte-colony stimulating factor (G-CSF) is known to improve healing of injured tissue. In addition, colistin is prescribed as an effective treatment. The aim of this study was to evaluate the effect of G-CSF and colistin alone or in combination with G-CSF on wound healing of *Acinetobacter baumannii* (*A. baumannii*) infected burns.

**Materials and Methods:**

This study was performed between January 2016 and April 2018. Burn wounds were experimentally induced in 36 mice. The wounds were inoculated with *A. baumannii*. In a 7-day period, burn wounds in each group were daily treated with subcutaneous injections (0.1 ml) of saline, G-CSF, colistin, and G-CSF plus colistin. After killing the animals, the size of the wound, number of leukocytes in the skin and microbial growth were evaluated. A value of p ≤ 0.05 was considered statistically significant.

**Results:**

Wound healing in the G-CSF plus colistin group was significantly higher than the control group and the G-CSF group (P = 0.023 and P = 0.033, respectively). In G-CSF+colistin group, the number of leukocytes was higher than the control group considerably (P = 0.007). On the 7^th^ day of treatment, number of positive bacterial cultures in the colistin and the G-CSF plus colistin groups was lower than other groups with a significant difference.

**Conclusion:**

Concurrent consumption of G-CSF and antibiotics can control burn infection and enhance the immune system towards wound healing.

## INTRODUCTION

Burns are always destructive and burn wounds are one of the most important health problems all over the world (1). There are several factors that can increase the chance of nosocomial infections in burn patients including characteristics of the disease, length of hospitalization, invasive diagnostic methods and therapeutic procedures and changes in the specific and nonspecific components of the immune system (2, 3). *Acinetobacter baumannii* is known as an important pathogen in nosocomial and burn infections in recent years (4). These bacteria have the tendency to develop resistance to multiple antibiotics rapidly (5). Today, multi-drug resistance in bacteria is a growing threat to public health world-wide and the widespread use of antibiotics has been associated with increase in resistant micro-organisms. Long-term consumption, irrational prescription and inappropriate dosing of antibiotics play an important role in appearance of resistance. Therefore, it is essential to find new therapeutic strategies for wound healing in infected burn injuries (6). Many cellular and immunological mechanisms are involved in wound healing processes, which include interactions between several growth factors, matrix proteins, their receptors and immune modulatory agents (7). The most important immunological change happening in burn wounds is the reduction of different neutrophil functions such as chemotaxis, phagocytes and oxidative burst. These changes disturb the process of wound healing in the burn (8).

Granulocyte-colony stimulating factor (G-CSF) is a cytokine, which has been used to reverse neutropenia in some cases such as catatonic chemotherapy and bone marrow and hematopoietic stem cell transplantation (9, 10). Studies show that G-CSF is effective in wound healing, and its actions directly affect the initial leukocyte-forming cells (11). It also improves neutrophil functions including chemotaxis (12). Colistin is used as a rescue therapy for nosocomial infections caused by multidrug-resistant (MDR) Gram-negative bacteria such as *A. baumanni* (13). Therefore, finding new therapeutic drugs and targets for wound healing in infected burn wounds is important. The aim of the present study was to evaluate the impact of Granulocyte-colony stimulating factor on wound healing in infected mice.

## MATERIALS AND METHODS

This study was performed in two steps, *in vitro* and *in vivo* and the following laboratory steps were carried out:

### *In vitro* step; Clinical isolates.

A total of 30 isolates of *A. baumannii* were collected from burn wounds in patients of Motahari Hospital in Tehran which is affiliated to the Iran University of Medical Sciences. The samples were identified using standard diagnostic biochemical tests.

### Antibiotic susceptibility testing.

Antimicrobial susceptibility and resistance were determined using disk diffusion method according to guidelines of the Clinical and Laboratory Standards Institute (CLSI) (14). Antibacterial susceptibility of *A. baumannii* strains was checked for 8 different antibiotic disks. These include ceftazidime (30 μg) gentamicin (10 μg), amikacin (30 μg), imipenem (10 μg), cefipime (30 μg), chloramphenicol (30 μg), ciprofloxacin (30 μg) and tetracycline (30 μg), (Mast Group Ltd., Merseyside, UK). In this study, *A. baumannii* ATCC 19606 was used as the reference strain for quality control.

Results were interpreted as susceptible, intermediate, or resistant according to the guidelines (Table 2). In the current study, a sample which had the highest antimicrobial resistance was selected for the *in-vivo* step. Antibiotic susceptibility was determined by the diameter of the inhibition of growth based on the CLSI guidelines.

### *In vivo* step; animal model.

Thirty six adult male Balb/c mice with similar age (18–20 month) and weight (20 ± 2g) were transferred from Pasteur Institute of Iran to animal laboratory of Iran University of Medical Sciences. Animals were healthy and did not have any particular illness. They were handled and taken care of, based on standard principles (15). The animals were maintained under controlled conditions such as 12 h light/12 h dark cycles, room temperature (32 ± 2°C) and relative humidity of 60–70%. They were given enough space to move freely and had access to food and water which were refilled every day. When general condition of the mice was deteriorated, they were excluded from the study.

Mice were randomly placed in 4 groups which had 9 members each (Table 1).

**Table 1.** Mice in different groups treated with different formulation.

**Groups (n = 9)**	**Interventions**
Control	subcutaneous injections of saline (0.1 ml) subcutaneous
Test I	injections of G-CSf treatment (10 μg/kg) subcutaneous
Test II	injections of colistin Antibiotics (50 mg/1kg)
Test III	subcutaneous injections of G-CSf treatment (10 μg/kg) and colistin antibiotics (50 mg/1kg)

### Induction of burn wounds and treatment procedure.

The mice were anesthetized using 10 mg/kg of ketamine and 4 mg/kg of Xylazine intraperitoneally. Afterwards, the dorsal area of the animals was shaved and disinfected by ethanol 70% (16). For induction of second degree burn, a hot device (95°C) comprised of a 2 cm in diameter circular steel rod was applied to the dorsal area for 8 seconds and then was covered with a sterilized gauze.

Twenty four hours after the induction of burns, the wounds were inoculated with 1 ml suspension of *A. baumannii* (1.5 × 10^8^ CFU/mL) and as it was reflected in Table 1, all animals were divided into 4 groups and injected with saline, G-CSF, colistin and G-CSF+colistin.

### Evaluation of improvement process in mice.

To evaluate the improvement process in mice, general conditions of the animal including weight, state of the immune system and also wound condition were investigated.

### General improvement of mice.

To assess the general conditions of the animals, all of them were weighed on the first and the last day of treatment process (17). The mean weight of all animals was calculated on the first and the last day. The mean was considered as a criterion for the general improvement. All animals in each group were weighed daily.

### Evaluation of the wound healing process.

All animals were killed by overdose of ether anesthetics on the 7^th^ (last) day of post treatment and the following evaluations were conducted:

### Morphological assessment.

Burn wounds were evaluated 24 hours after the induction (after the burn/before the treatment: first day) and on the 7^th^ day (after the treatment: last day) by measuring the area of the wounds with the naked eye (18). The percentage of wound recovery was calculated implementing the following formula:

$$\begin{array}{l} \begin{array}{l} \text{Wound}\;\text{area}\;\text{on}\;\text{first}\;\text{day}\;({\text{mm}}^{2})-\text{wound}\;\text{area}\;\text{on}\\ \text{last}\;\text{day}\;({\text{mm}}^{2})/\text{wound}\;\text{area}\;\text{on}\;\text{first}\;\text{day}\;({\text{mm}}^{2})\times 100 \end{array} \end{array}$$

### Hematological assessment.

To survey of condition of the immune system, immune cells were counted (19). Therefore blood samples were collected using heparin coated tubes. Leukocytes were counted on the 7^th^ day of treatment by a cell counting device (cell Counter Refreshed Sysmex kx2).

### Bacterial assessment.

On the 7^th^ day of treatment, the surface layer of the lesion was removed by a wet sterile swab in all mice and was cultured on blood agar medium and incubated at 37°C. The cultures were checked for *A. baumannii* using colony morphology, microscopic Gram-stain investigation and standard biochemical tests after 24 h (20).

### Statistical analysis.

The statistical analysis was performed with SPSS software, version 20.0. Animal group comparisons were done by one-way analysis of variance (ANOVA) and were followed by Tukey–Kramer test. A value of p≤0.05 was considered to be statistically significant.

## RESULTS

Evaluation of antimicrobial susceptibility and resistance the antibiotic resistance patterns of 30 clinical samples of *A. bumannii* are shown in Table 2.

**Table 2.** The percentage of resistant isolates (Total population = 30)

**Antibiotic**	***Acinetobacter bumanni*** **Resistant N (%)**
Ceftazidime (30 μg)	30 (100)
Gentamicin (10 μg)	26 (86.7)
Amikacin (30 μg)	30 (100)
Imipenem (10 μg)	30 (100)
Cefipime (30 μg)	30 (100)
Ciprofloxacin (30 μg)	30 (100)
Tetracycline (30 μg)	22 (73.4)

### General improvement.

**Fig. 1.**
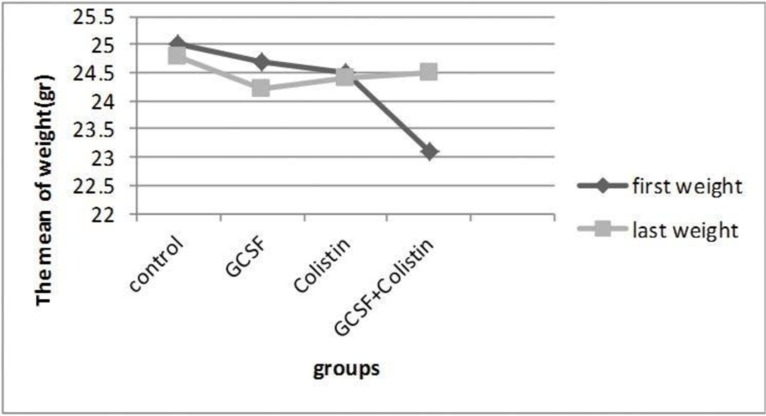
Comparison of the mean weights of all groups

In order to determine the amount of general improvement each mouse had experienced, all animals were weighed daily (based on gr) in each group. The mean weight of the first and the last day were calculated in each group and were compared between the studied groups. Result showed that in G-CSF+colistin group, the mean of weight of animals on the last day was higher than the mean of weight of them on the first day but not significant (Fig. 1).

### Morphology examination of the wounds.

Burn wounds were evaluated starting 24 hours after the induction of burn and also on the last day after treatment. Percentage of wound recovery in all treated groups was compared to other groups (Table 3). The results showed that the percentage of wound recovery in G-CSF+colistin group was significantly higher than the control group (P = 0.023)* and was also considerably greater than the G-CSF group (P = 0.033)* compared to all other groups. In this study the statistical method of one-way analysis of variance (ANOVA) followed by Tukey–Kramer test (F; 3.98, df: 3, sig: 0.018) was used.

**Table 3.** The percentage of overall wound recovery measured on the 7^th^ day of treatment in 4 groups (mean ± SD).

The percentage of wound recovery	
Groups	mean ± SD
Control	13.14±15.27
G-CSF	13.72±15.59
Colistin	21.60±17.32
G-CSF+colistin	38.87±20.32

### White blood cell assessment.

**Fig. 2.**
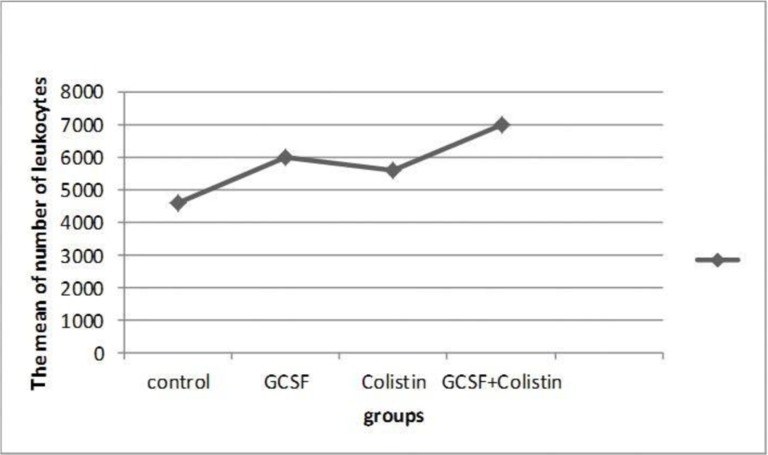
Comparative study of leukocytes in all groups.

As Fig. 2 indicates, the number of leukocytes in G-CSF group was higher than the control group but not significantly, however, in G-CSF+colistin group, the number of leukocytes was significantly higher than the control group (P = 0.007)*. In this study the statistical method of one-way analysis of variance (ANOVA) followed by Tukey–Kramer test (F: 6.85, df: 3, sig: 0.01) was used.

### Bacterial assay.

**Fig. 3.**
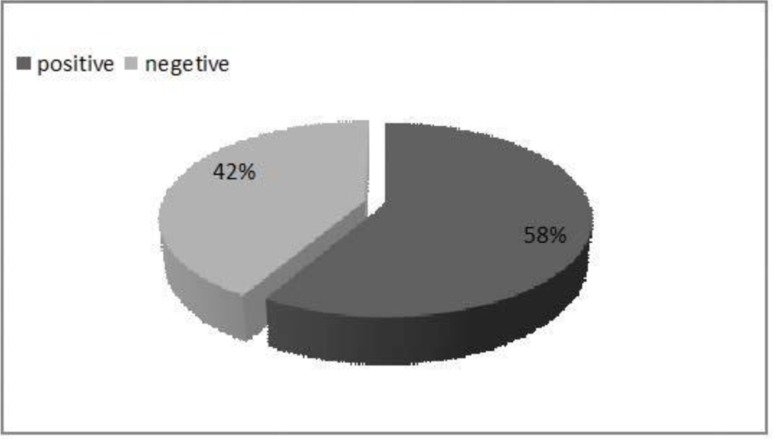
Percentages of bacterial from swabs

On the last day of treatment, the surfaces of the lesions were removed and cultured and then were checked for *A. baumannii* after 24 h. Percentage of positive and negative bacterial cultures were calculated for all groups simultaneously (Fig. 3) and also separately for each group (Table 4). From the total number of the culture swabs (n=36), 21 (58%) were positive and 15 (42%) were negative for bacteria (Fig. 3). In evaluation of the burn wound swabs, percentage of the cases with no bacterial growth was calculated for each group (Table 4).

**Table 4.** The percentage of the cases negative bacterial culture in each group after burn induction in 7^th^ day of treatment

The percentage of the cases negative bacterial culture	
Groups	Percentage
Control	22
G-CSF	0
Colistin	100
G-CSF+colistin	100

After injury induction, percentage of the cases negative in bacterial culture calculated in each group in last day of treatment. This percentage among groups has significant difference (by statistical method Npar tests, Kruskal Wallis (Chi-square:221.00, df: 2, sig: 0.000)).

## DISCUSSION

In this study all the *A. bumannii* isolates were resistant to imipenem, cefipime, ciprofloxacin, ceftazidime and amikacin (Table 2). *Acinetobacter spp* develop resistance to new antimicrobial drugs quickly and can gain resistance faster than other Gram-negative organisms.

Diseases and infections can weaken or impair the immune system. One of the consequences of a defective immune system is malnutrition which eventually leads to weight loss (21, 22). The results of some studies showed relationship between the immune system and the weight (21, 22), so the immune system boosters may affect the weight. Actually we expected that when the immune system was strengthened (in recipient groups of G-CSF) the weight of the animal increased. However, in our study there was no considerable difference between the final and the initial weights of each group (Fig. 2), with the exception of G-CSF+colistin group in which the eventual weight had risen, although not significantly.

In the current study, it was found that percentage of wound recovery after seven days, was significantly higher when G-CSF and colistin were used concurrently comparing to the control group (P = 0.023)* and the G-CSF group (P = 0.033)* in Table 3. It seems that the percentage of wound recovery increased after seven days in the G-CSF+colistin group.

The new therapeutic methods improve the immune system and remove the infection for wound healing. G-CSF can help in the wound healing by reducing translocation and colonization of the bacteria, and increasing blood neutrophils (23, 24). In addition, G-CSF can increase the number of neutrophils and macrophages (25). Yet the role of neutrophils in wound healing is questionable (23). The result of a study in 2008 showed that G-CSF increases neutrophil recruitment and reduces the time needed for wound healing (20). Also in a study that was conducted by Aleah L. Brubaker et al. in 2013, it was found that wound closure was enhanced in the aged mice receiving G-CSF (23). Moreover colistin is only drug universally active against multi resistant clinical strains (25, 26). The results of a study in 2016 on patients showed that in the group of receiving colistin, microbiologic failure rates were higher at Seventh day of treatment (25). Actually colistin can enhance bacterial clearance and wound closure (23–27). Overall, according to this study, the use of G-CSF and colistin simultaneously can be more effective in wound recovery.

G-CSF is a glycoprotein which is produced by monocytes and fibroblast cells. It is a granulocyte simulator which induces the growth of granulocyte precursors, increases the number of white blood cells and also neutrophil activity. One of the causes of bacterial infection in burns is the malfunction of neutrophils. Therefore, prescribing G-CSF may be beneficial (23, 27, 28). In this research, it was revealed that after seven days of solely using G-CSF, the numbers of leukocytes were increased (but not significantly) in G-CSF group compared to the control group. In a similar study, Brubaker et al, expressed that G-CSF can be involved in wound closure by increasing wound neutrophil recruitment (23). G-CSF also significantly augmented the number of leukocyte in rat in another study (28). In our project there was a rise in the number of leukocyte when antibiotics (colistin) were used but it was lower than group that took G-CSF. It seems that G-CSF and antibiotics, when used simultaneously, act synergistically and, the concurrent use of them results in a significant increase in the number of leukocytes (P = 0.007) compared to the control group (Fig. 3). Increase in the activity of human neutrophil elastase by colistin has recently been reported (29, 30). But study on the combined effect of G-CSF and colistin needs to be investigated. Short term consumption of antibiotics reduces the bacterial infections and stimulates the immune system and also can relatively increase the immunoglobulin and leukocytes in the blood. On the other hand, using antibiotics for a long period can weaken the immune system and lead to development of antibiotic resistance (31–32). Overall, antibiotics directly take part in the removal of infections by increasing and improving immune cells while G-CSF helps indirectly. Therefore, the concurrent consumption of G-CSF and antibiotics can control the infection and improve the immune system. In this study, 58% of bacterial cultures which were obtained from burn wounds on the last day of treatment were positive for *A. baumannii* (Fig. 4). Bacterial infections in burn wounds might appear to have a higher prevalence rather than other forms of trauma, because skin is the first line of defense against infections and is the place where the first alterations of cellular and humoral immune responses happen (33). All mice in colistin and G-CSF+colistin groups showed negative bacterial growth on 7^th^ day of treatment after the injury induction (sig: 0.000)* (in table 4). In this regard, in the project of Brubaker et al. conducted on BALB/c mice, the enhancement of bacterial clearance in G-CSF was confirmed (23). The results indicate that treating burn wounds with colistin and G-CSF+colistin may decrease or eliminate bacterial infection. However, G-CSF alone may not be enough to eliminate the infection.

In conclusion, G-CSF can be effective in wound closure by controling the infection and improving the immune system. In fact, combination of antibiotic and G-CSF if used appropriately can control the infection through colistin and improving the immune system through G-CSF.
